# TBS Predict Coronary Artery Calcification in Adults

**DOI:** 10.1155/2016/8391589

**Published:** 2016-03-06

**Authors:** Tzyy-Ling Chuang, Fu-Tsung Hsiao, Yi-Da Li, Yuh-Feng Wang

**Affiliations:** ^1^Department of Nuclear Medicine, Dalin Tzu Chi Hospital, Buddhist Tzu Chi Medical Foundation, Chiayi 62247, Taiwan; ^2^Department of Medical Imaging, Dalin Tzu Chi Hospital, Buddhist Tzu Chi Medical Foundation, Chiayi 62247, Taiwan; ^3^Department of Cardiology, Dalin Tzu Chi Hospital, Buddhist Tzu Chi Medical Foundation, Chiayi 62247, Taiwan; ^4^School of Medicine, Tzu Chi University, Hualien, Taiwan

## Abstract

*Purpose*. This study analyzes the association between the bony microarchitecture score (trabecular bone score, TBS) and coronary artery calcification (CAC) in adults undergoing health exams.* Materials and Methods*. We retrospectively collected subjects (*N* = 81) who underwent coronary computed tomography and bone mineral density studies simultaneously. CAC was categorized to three levels (Group 0, G0, no CAC, score = 0, *N* = 45; Group 1, G1, moderate CAC, score = 1–100, *N* = 17; Group 2, G2, high CAC, score ≧ 101, *N* = 19). Multinomial logistic regression was used to study the association between TBS and CAC levels.* Results*. CAC is present in 44.4% of the population. Mean TBS ± SD was 1.399 ± 0.090. Per 1 SD increase in TBS, the unadjusted odds ratio (2.393) of moderate CAC compared with no CAC was significantly increased (95% CI, 1.219–4.696, *p* = 0.011). However, there has been no association of TBS with high CAC (OR: 1.026, 95% CI: 0.586–1.797, *p* = 0.928). These relationships also existed when individually adjusted for age, sex, and multiple other covariates.* Conclusions*. Higher TBS was related to moderate CAC, but not high CAC; a possible explanation may be that bone microarchitecture remodeling becomes more active when early coronary artery calcification occurs. However, further researches are needed to clarify this pathophysiology.

## 1. Introduction

Atherosclerosis and osteoporosis share many risk factors, but their independent association is unclear [1]. The diagnosis of osteoporosis depends on area bone mineral density (BMD) measurements using dual energy X-ray absorptiometry (DXA) [2]. BMD has been inversely associated with subclinical and clinical cardiovascular disease (CVD), even after adjusting for potential confounding factors [3]. Previous studies have examined the relationship between trabecular volumetric BMD (vBMD) and aortic arterial calcification (AAC) or coronary artery calcification (CAC), with inconclusive results [3–6]. Cortical, but not trabecular, vBMD was associated with significantly decreased odds of AAC prevalence independent of other traditional risk factors [3]. With a decrease in vBMD, the adjusted odds of high AAC, compared with no AAC, were significantly increased; vBMD was related to high CAC in unadjusted, but not adjusted, models. No associations of vBMD with moderate AC or CAC were observed [5].

Practically, BMD evaluated by DXA studies was a presentation of both cortical and trabecular bone content. However, cancellous bone microarchitecture is the key determinant of bone strength, which is often measured by quantitative computed tomography (qCT). But this involves higher radiation exposure, is more expensive, and has a larger instrument requirement. TBS (trabecular bone score) is a texture parameter that can be computed from the two-dimensional lumbar spine DXA image [7]. TBS, a variogram, is related to bone microarchitecture (few large spans, i.e., low TBS, are mechanically weaker than a myriad of fine spans, i.e., high TBS) and is complementary to predict fracture risk, as well as lumbar spine BMD measurements. Therapeutic strategies for osteoporosis differ after inclusion of the influence from TBS [7].

The clinical application of TBS and the association of CAD have not been documented. The aim of this study is to explore the relationship of TBS and CAC in adults undergoing a health exam.

## 2. Materials and Methods

### 2.1. Subjects

We retrospectively collected patients who had simultaneously undergone a coronary CT scan and BMD study from May 2014 to November 2015, after the introduction of the DXA equipment (HOLOGIC Discovery Wi) in the health examination center at Dalin Tzu Chi Hospital. The interval between the two tests varied from the same day to one month.

Health history (by interview or questionnaire), anthropomorphic characteristics, and laboratory data, including lipid profile (total cholesterol, LDL, HDL, and triglyceride), glucose levels, systolic and diastolic blood pressure, and smoking history, were recorded (Table 1).

### 2.2. Coronary Artery Calcification

CAC scoring was obtained on unenhanced axial images scanned before the coronary CT angiography. The scans were performed using a multidetector CT system (LightSpeed VCT, GE Medical Systems). CAC was quantified with the Agatston scoring method, which has been widely accepted [8]. Total calcium score was determined using the sum of individual scores from the four major coronary arteries (left main, left anterior descending, circumflex, and right coronary arteries).

### 2.3. TBS Measurement

TBS (trabecular bone score), a texture parameter, is computed from the DXA images. TBS can be quantified from local variations in pixels intensities and derived from the experimental variogram obtained from the gray levels of a DXA image. With TBS iNsight installed on the DXA device PC, it quantified the bone texture in 3 s by retrospectively automatic analysis from an existing DXA scan without additional examination or dosage for the patient.

### 2.4. Statistical Analysis

45 subjects (54.9%) had a CAC score of zero and small sample size, so the CAC data analysis was treated as categorical 0 (CAC = 0), 1 (0 < CAC ≤ 100), and 2 (CAC > 100).

Differences in means or frequencies between characteristics statuses were tested by chi-squared test or ANOVA, as appropriate. Multinomial logistic regression was used to identify the significant predictors of coronary artery calcification after adjustment for other cofactors. Models of TBS predicting CAC were developed through addition of covariates to assess the strength and independence of the associations.

Covariates included age, sex, hypertension, diabetes, hyperlipidemia, systolic blood pressure, diastolic blood pressure, and measured laboratory data (total cholesterol, LDL, HDL, triglyceride, and glucose). Odds ratios were expressed as the effect of a 1 SD or unit increase in covariate or TBS in adjusted, unadjusted, or age-adjusted models.

Statistical analysis was performed using PASW Statistics 18 (SPSS Inc., Chicago, IL).

## 3. Results

### 3.1. Participant Characteristics

**Figure 1:**
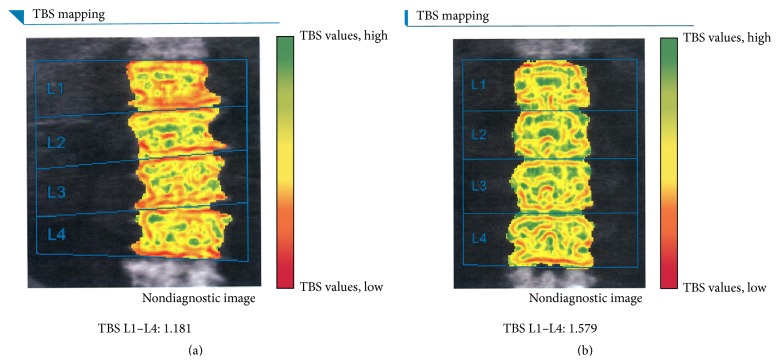
(a) A case in group 2 (high CAC), a 69-year-old female with hypertension and hyperlipidemia, height 151.0 cm, and weight 51.0 kg. CAC score is 1185; TBS value of L1–L4 showed 1.181. (b) A case in group 1 (moderate CAC), a 51-year-old male without any systemic disease, height 163.0 cm, and weight 68.0 kg. CAC score is 38; TBS value of L1–L4 showed 1.579.

Total 81 subjects (51 males and 30 females) were collected from our database. Out of them, 36 (44.4%) had coronary artery calcification, described as CAC > 0. The average TBS was 1.40 ± 0.09 (SD). Participants with high CAC were older and more likely to be hypertensive, compared to those with moderate or no CAC. A significant increase in glucose and TBS was observed with moderate CAC, as compared with the no CAC group (Table 1). In our cohort, no one had chronic kidney disease. One case had bilateral total hip replacement and was not included in the final analysis. An example of group 2 and another case from group 1 with their TBS figures were shown in Figure 1.

### 3.2. Predictors of CAC

A 9.92-year (1 SD) greater was associated with 2.87 times greater odds of high CAC, as compared with no CAC (Table 2). Male gender and diabetes after age-adjusted significantly increased the odds (4.18 and 21.02 times) of moderate CAC, relative to the no CAC group. Hypertensive subjects had 8.05 times greater age-adjusted odds in high CAC than in no CAC. Hyperlipidemia, SBP, DBP, total cholesterol, LDL, HDL, triglyceride, and glucose were not significantly associated with moderate CAC or high CAC after adjustment for age.

### 3.3. TBS and CAC

In unadjusted multinomial logistic regression analysis, per 1 SD increase in TBS, the odds of moderate CAC compared with no CAC were significantly increased 2.39-fold (95% CI, 1.22–4.70). The association remained significant after individually adjusting of age, sex, hypertension, diabetes, hyperlipidemia, systolic blood pressure (SBP), diastolic blood pressure (DBP), lipid profile, and glucose and after each combination adjustment for age and sex, SBP and DBP, even in an extensive adjusted model, which included age, LDL, SBP, and glucose. However, no significant relationship was observed between TBS and high CAC in unadjusted or adjusted analyses (Table 3).

## 4. Discussion

There is a link between osteoporosis and cardiovascular disease (CVD) [5]. Subjects who self-report a previous myocardial infarction had significantly higher odds of having low bone mineral density, when adjusting for CVD and osteoporosis risk factors, and this was not significantly associated in women but was significant in men [9]. Postmenopausal women with osteoporosis are at an increased risk for cardiovascular events, proportional to the severity of osteoporosis at the time of the diagnosis [10].

CAC had the role in developing CAD [11–13]; CAC and CT angiography in asymptomatic elderly patients can predict coronary artery disease [14]. Some studies showed a negative association between BMD (or vBMD) and score or presence of aortic calcification (AC)/coronary artery calcification (CAC) [5, 15, 16]. Their relationship may be age-related progression [3], shared risk factors (smoking), or common pathophysiological mechanisms (hormones or inflammatory cytokines) [5]. The association between cortical BMD (not trabecular vBMD) and AAC persisted even after adjustment for age, BMI, lifestyle factors, diabetes, and hypertension [3], while other studies showed an association of trabecular vBMD with AAC [5]. Their inconsistency with regard to results may be due to sex- and/or ethnicity-specific differences [3, 5]. Cortical and trabecular bone are known to have different turnover rates and age-related patterns [17]. The strongest predictors of AAC prevalence include increased age, male sex, smoking, higher BMI or waist circumference, hypertension, dyslipidemia, and diabetes [3, 18, 19].

In a study of volumetric BMD and vascular calcification measured by CT in middle-aged women, they divided the population of AC and CAC into three levels and found that lower trabecular BMD of the spine was significantly associated with high AC levels and also high CAC levels; the latter was not significant after adjusting for age [5]. In a recent Rotterdam Study, no association between CAC and BMD or fracture risk was found, except for BMD loss with higher follow-up CAC in women, which may be related to low estrogen levels [6].

Vascular calcifications (VCs) are of similar composition to bone minerals. Currently, intima-related VCs are commonly associated with atherosclerotic plaques (in the vicinity of lipid or cholesterol deposits) and lesions calcified lately, and lesions of media-related VCs calcified early (in the absence of lipid or cholesterol deposits) [20]. Even if medial and intimal calcification may share some common pathomechanisms and can occur together in patients, it is reasonable to maintain a distinction between the two [21]. VCs represent complex biological process of calcium phosphate deposition and are related to regulation of osteogene expression, bone morphogenetic protein (BMP2), calcification inhibitors (osteoprotegerin, matrix-gla protein, fetuin-A), and inflammatory cytokines (TNF-*α*, CRP, and CD40–CD154) [20, 21]. Unfortunately, it is impossible, or at least extremely difficult, to distinguish between intimal and medial calcification in the coronary arteries [21].

The absence of CAC strongly excludes obstructive CAD, and CAC predicts the presence of coronary atherosclerotic plaque. However, the absence of any CAC does not exclude the presence of coronary atherosclerotic plaque, especially in patients aged <55 years. Plaque composition shifted from noncalcified to calcified plaque with increasing age, which may affect the vulnerability of these lesions over time [22]. CAC has also been associated with high serum concentration of some biomarkers, including undercarboxylated osteocalcin and fibroblast growth factor 23 [23, 24]. In patients on dialysis, high parathyroid hormone level and osteoporosis predict progression of CAC [25]; and bone volume/total volume (BV/TV) assessed by HR-pQCT were significantly lower in patients with CAC scores ≥100 [26].

The BMD *T*-score may not fully capture the fragility fracture risk, so the noninvasive analytic tool of TBS was developed. The TBS is a texture parameter that evaluates pixel gray-level variations in DXA images of the lumbar spine [2]. TBS decreases with age and appears to reflect qualitative aspects of skeletal structure complementary to BMD [7]. Quantitative computed tomography has the disadvantage of higher radiation exposure, increased expense, and larger instrument requirements. TBS measure the trabecular microarchitecture with simple DXA machine [27], which is cheaper, involves less radiation exposure, and only needs an immediately “1 click – 3 s” extra software analysis to the traditional lumbar spine BMD data, without additional exams or radiation doses. The TBS also can be retrospectively analyzed in the same machine from an existing DXA scan to quantify bone microarchitectural texture.

Our result suggested that TBS value (per 1 SD increase) positively predicted the group of moderate CAC (odds ratio = 2.39, *p* = 0.011) but had no association for the high CAC group (odds ratio = 1.03, *p* = 0.928). The relationship still existed even after adjusting for the covariates. This result is significantly different from previous studies with qCT and CAC [1, 26]. The difference might be possible due to the diverse methodologies. Since VCs have complex mechanisms, another possible explanation may be that early CAC is associated with a more complex variogram of bone microarchitecture during bone remodeling. However, at far-advanced CAC, the higher TBS had no significance in prediction value. It means that molecular cascades and procalcific microenvironment during “vascular calcification dynamics” change with the process of “bone microarchitecture formation.” During early CAC, both are similar. In severe CAC, the direction of the kinetic equilibrium is stable and the progression of evolution makes the relationship between CAC and TBS not develop further. Exposure to high Ca concentrations may influence the development of low-turnover bone disease and coronary artery calcification (CAC) in patients on hemodialysis (HD) [28]. For cases under DXA measurement of lumbar spines, the BMD value may be overestimated for the cases with abdominal aorta calcification [29]. Although, in our CAC group 1, the mean TBS was higher than group 0, in our CAC group 2, the mean TBS was lower than CAC group 1. The projection interference, a potential confounder, may not be a factor that influences our results.

## 5. Conclusion

Atherosclerosis and vascular calcification are dynamic processes; both of them and bone microarchitecture reach a dynamic equilibrium in bone remodeling. Advanced age is significantly associated with high CAC (score > 100), while increased TBS is associated with moderate CAC (0 < score ≤ 100), independent of age and other risk factors. These unusual findings are most likely due to the deferent biomechanism of diverse methodology or complex regulatory networks of VCs and need further research and a larger database for confirmation.

## Figures and Tables

**Table 1:** Participants characteristics.

	No CAC (G0)	Moderate CAC (G1)	High CAC (G2)	*p*
	(Score = 0)	(Score = 1–100)	(Score ≧ 101)	
	(*N* = 45)	(*N* = 17)	(*N* = 19)	
Age (years)	53.9 ± 9.9^a^	53.6 ± 6.3^a^	62.3 ± 10.1^b^	0.004
Female (%)	21 (70)	3 (17.6)	6 (31.6)	0.092
Smoking (%)	4 (8.9)	1 (5.9)	1 (5.3)	0.848
HTN (%)	6 (13.3)	1 (5.9)	12 (63.2)	<0.001
DM (%)	1 (2.2)	5 (29.4)	3 (15.8)	0.007
HL (%)	2 (4.4)	1 (5.9)	2 (10.5)	0.652
Weight (kg)	66.5 ± 12.5	72.6 ± 13.7	67.1 ± 9.7	0.206
Height (cm)	163.3 ± 8.3	168.0 ± 9.0	162.1 ± 6.1	0.064
TCH (mg/dL)	191.8 ± 35.7	178.4 ± 26.1	192.3 ± 29.8	0.317
LDL (mg/dL)	125.0 ± 29.8	112.7 ± 19.4	121.4 ± 27.6	0.291
HDL (mg/dL)	51.1 ± 16.5	43.4 ± 7.9	47.4 ± 15.2	0.178
TG (mg/dL)	130.4 ± 70.6	175.1 ± 75.5	153.5 ± 147.6	0.237
Glucose (mg/dL)	100.8 ± 10.2^a^	124.3 ± 32.8^b^	107.8 ± 20.8^a^	<0.001
SBP (mmHg)	124.1 ± 24.8	124.4 ± 14.0	132.1 ± 24.8	0.427
DBP (mmHg)	77.4 ± 18.1	77.4 ± 9.8	83.0 ± 11.8	0.394
TBS	1.384 ± 0.083^a^	1.451 ± 0.081^b^	1.386 ± 0.101^ab^	0.024

Note: means with different superscripts indicate significant difference at *p* < 0.05 level, evaluated using Sidak post hoc adjustment.

HTN: hypertension; DM: diabetes mellitus; HL: hyperlipidemia; TCH: total cholesterol; LDL: low-density lipoprotein; HDL: high-density lipoprotein; TG: triglyceride; SBP: systolic blood pressure; DBP: diastolic blood pressure; TBS: trabecular bone score.

**Table 2:** Odds of age-adjusted covariates at the multinomial logistic regression model for CAC.

Covariates	CAC group		
	Age-adjusted OR (95% CI)		
	No CAC (G0)	Moderate CAC (G1)	High CAC (G2)
Age^*∗*^	1.00	0.96 (0.53–1.74) [0.896]	2.87 (1.42–5.81) [0.003]
Male versus female	1.00	4.18 (1.04–16.83) [0.044]	2.31 (0.68–7.89) [0.181]
HTN	1.00	0.41 (0.05–3.71) [0.425]	8.05 (2.13–30.41) [0.002]
DM	1.00	21.02 (2.15–205.62) [0.009]	6.13 (0.56–67.25) [0.138]
Hyperlipidemia	1.00	1.39 (0.11–16.85) [0.797]	1.78 (0.21–14.89) [0.596]
SBP^*∗*^	1.00	1.02 (0.57–1.81) [0.960]	1.31 (0.73–2.35) [0.375]
DBP^*∗*^	1.00	1.00 (0.55–1.83) [0.988]	1.36 (0.76–2.44) [0.308]
TCH^*∗*^	1.00	0.65 (0.36–1.18) [0.156]	1.02 (0.57–1.82) [0.958]
LDL^*∗*^	1.00	0.64 (0.36–1.14) [0.129]	0.82 (0.45–1.52) [0.535]
HDL^*∗*^	1.00	0.54 [0.27–1.08] [0.081]	0.70 (0.38–1.27) [0.081]
TG^*∗*^	1.00	1.67 (0.90–3.10) [0.102]	1.72 (0.89–3.33) [0.108]
Glucose^*∗*^	1.00	3.53 (1.59–7.85) [0.002]	1.90 (0.83–4.33) [0.129]

Values are odds ratios (95% CI) [*p* value].

^*∗*^For 1 SD increase in age (9.92 y/o), SBP (22.93 mmHg), DBP (15.35 mmHg), TCH (32.68 mg/dL), LDL (27.53 mg/dL), HDL (14.96 mg/dL), and TG (95.47 mg/dL)

HTN: hypertension; DM: diabetes mellitus; SBP: systolic blood pressure; DBP: diastolic blood pressure; TCH: total cholesterol; LDL: low-density lipoprotein; HDL: high-density lipoprotein; TG: triglyceride.

**Table 3:** Odds of TBS with or without adjustment at the multinomial logistic regression model for CAC.

Covariates	No CAC (G0)	Moderate CAC (G1)	High CAC (G2)
TBS (unadjusted)^*∗*^	1.00	2.39 (1.22–4.70) [0.011]	1.03 (0.59–1.80) [0.928]
TBS (adjusted for age)^*∗*^	1.00	2.60 (1.28–5.29) [0.008]	1.63 (0.836–3.173) [0.152]
TBS (adjusted for sex)^*∗*^	1.00	2.07 (1.02–4.20) [0.044]	0.89 (0.48–1.65) [0.707]
TBS (adjusted for age and sex)^*∗*^	1.00	2.27 (1.07–4.78) [0.032]	1.45 (0.70–3.04) [0.320]
TBS (adjusted for HTN)^*∗*^	1.00	2.43 (1.20–4.90) [0.014]	1.53 (0.78–3.00) [0.214]
TBS (adjusted for DM)^*∗*^	1.00	2.63 (1.26–5.48) [0.010]	1.03 (0.58–1.82) [0.925]
TBS (adjusted for hyperlipidemia)^*∗*^	1.00	2.43 (1.22–4.82) [0.011]	1.03 (0.59–1.79) [0.923]
TBS (adjusted for SBP)^*∗*^	1.00	2.47 (1.24–4.89) [0.010]	1.14 (0.63–2.04) [0.670]
TBS (adjusted for DBP)^*∗*^	1.00	2.42 (1.23–4.75) [0.011]	1.05 (0.60–1.84) [0.857]
TBS (adjusted for SBP and DBP)^*∗*^	1.00	2.46 (1.24–4.88) [0.010]	1.12 (0.62–2.02) [0.700]
TBS (adjusted for smoking)^*∗*^	1.00	2.41 (1.23–4.74) [0.011]	1.03 (0.59–1.80) [0.918]
TBS (adjusted for TCH, LDL, HDL, and TG)^*∗*^	1.00	2.30 (1.12–4.74) [0.023]	0.90(0.48–1.68) [0.734]
TBS (adjusted for glucose)^*∗*^	1.00	2.52 (1.16–5.45) [0.019]	1.02 (0.58–1.80) [0.949]
TBS (adjusted for age, LDL, SBP, and glucose)^*∗*^	1.00	2.71 (1.20–6.12) [0.016]	1.73 (0.85–3.50) [0.128]

Values are odds ratios (95% CI) [*p* value].

^*∗*^For 1 SD increase in TBS.

TBS: trabecular bone score; HTN: hypertension; DM: diabetes mellitus; SBP: systolic blood pressure; DBP: diastolic blood pressure; TCH: total cholesterol; LDL: low-density lipoprotein; HDL: high-density lipoprotein; TG: triglyceride.

## References

[1] Hyder J. A., Allison M. A., Wong N. (2009). Association of coronary artery and aortic calcium with lumbar bone density: the MESA Abdominal Aortic Calcium Study. *American Journal of Epidemiology*.

[2] Bousson V., Bergot C., Sutter B., Levitz P., Cortet B. (2012). Trabecular bone score (TBS): available knowledge, clinical relevance, and future prospects. *Osteoporosis International*.

[3] Kuipers A. L., Zmuda J. M., Carr J. J. (2014). Association of volumetric bone mineral density with abdominal aortic calcification in African ancestry men. *Osteoporosis International*.

[4] Chow J. T., Khosla S., Melton L. J., Atkinson E. J., Camp J. J., Kearns A. E. (2008). Abdominal aortic calcification, BMD, and bone microstructure: a population-based study. *Journal of Bone and Mineral Research*.

[5] Farhat G. N., Cauley J. A., Matthews K. A. (2006). Volumetric BMD and vascular calcification in middle-aged women: the study of women's health across the nation. *Journal of Bone and Mineral Research*.

[6] Campos-Obando N., Kavousi M., Roeters van Lennep J. E. (2015). Bone health and coronary artery calcification: the Rotterdam Study. *Atherosclerosis*.

[7] Silva B. C., Leslie W. D., Resch H. (2014). Trabecular bone score: a noninvasive analytical method based upon the DXA image. *Journal of Bone and Mineral Research*.

[8] Agatston A. S., Janowitz W. R., Hildner F. J., Zusmer N. R., Viamonte M., Detrano R. (1990). Quantification of coronary artery calcium using ultrafast computed tomography. *Journal of the American College of Cardiology*.

[9] Magnus J. H., Broussard D. L. (2005). Relationship between bone mineral density and myocardial infarction in US adults. *Osteoporosis International*.

[10] Tankó L. B., Christiansen C., Cox D. A., Geiger M. J., McNabb M. A., Cummings S. R. (2005). Relationship between osteoporosis and cardiovascular disease in postmenopausal women. *Journal of Bone and Mineral Research*.

[11] Russo V., Zavalloni A., Reggiani M. L. B. (2010). Incremental prognostic value of coronary CT angiography in patients with suspected coronary artery disease. *Circulation: Cardiovascular Imaging*.

[12] Wexler L., Brundage B., Crouse J. (1996). Coronary artery calcification: pathophysiology, epidemiology, imaging methods, and clinical implications. A statement for health professionals from the American Heart Association. *Circulation*.

[13] Raggi P., Shaw L. J., Berman D. S., Callister T. Q. (2004). Prognostic value of coronary artery calcium screening in subjects with and without diabetes. *Journal of the American College of Cardiology*.

[14] Imanzadeh A., George E., Kondo T. (2016). Coronary artery calcium score and CT angiography in asymptomatic elderly patients with high pretest probability for coronary artery disease. *Japanese Journal of Radiology*.

[15] Schulz E., Arfai K., Liu X., Sayre J., Gilsanz V. (2004). Aortic calcification and the risk of osteoporosis and fractures. *Journal of Clinical Endocrinology and Metabolism*.

[16] Barengolts E. I., Herman M., Kukreja S. C., Kouznetsova T., Lin C., Chomka E. V. (1998). Osteoporosis and coronary atherosclerosis in asymptomatic postmenopausal women. *Calcified Tissue International*.

[17] Gabet Y., Bab I. (2011). Microarchitectural changes in the aging skeleton. *Current Osteoporosis Reports*.

[18] Liu J., Fox C. S., Hickson D. (2010). Pericardial adipose tissue, atherosclerosis, and cardiovascular disease risk factors: the Jackson heart study. *Diabetes Care*.

[19] Kuller L. H., Matthews K. A., Sutton-Tyrrell K., Edmundowicz D., Bunker C. H. (1999). Coronary and aortic calcification among women 8 years after menopause and their premenopausal risk factors: the Healthy Women study. *Arteriosclerosis, Thrombosis, and Vascular Biology*.

[20] Lanzer P., Boehm M., Sorribas V. (2014). Medial vascular calcification revisited: review and perspectives. *European Heart Journal*.

[21] Amann K. (2008). Media calcification and intima calcification are distinct entities in chronic kidney disease. *Clinical Journal of the American Society of Nephrology*.

[22] Choi T.-Y., Li D., Nasir K. (2013). Differences in coronary atherosclerotic plaque burden and composition according to increasing age on computed tomography angiography. *Academic Radiology*.

[23] Choi B. H., Joo N. S., Kim M. J., Kim K. M., Park K. C., Kim Y. S. (2015). Coronary artery calcification is associated with high serum concentration of undercarboxylated osteocalcin in asymptomatic Korean men. *Clinical Endocrinology*.

[24] Freedman B. I., Divers J., Russell G. B. (2015). Plasma FGF23 and calcified atherosclerotic plaque in African Americans with type 2 diabetes mellitus. *American Journal of Nephrology*.

[25] Malluche H. H., Blomquist G., Monier-Faugere M., Cantor T. L., Davenport D. L. (2015). High parathyroid hormone level and osteoporosis predict progression of coronary artery calcification in patients on dialysis. *Journal of the American Society of Nephrology*.

[26] Cejka D., Weber M., Diarra D., Reiter T., Kainberger F., Haas M. (2014). Inverse association between bone microarchitecture assessed by HR-pQCT and coronary artery calcification in patients with end-stage renal disease. *Bone*.

[27] Winzenrieth R., Michelet F., Hans D. (2013). Three-dimensional (3D) microarchitecture correlations with 2D projection image gray-level variations assessed by trabecular bone score using high-resolution computed tomographic acquisitions: effects of resolution and noise. *Journal of Clinical Densitometry*.

[28] Ok E., Asci G., Bayraktaroglu S. (2015). Reduction of dialysate calcium level reduces progression of coronary artery calcification and improves low bone turnover in patients on hemodialysis. *Journal of the American Society of Nephrology*.

[29] Demer L. L. (2002). Vascular calcification and osteoporosis: inflammatory responses to oxidized lipids. *International Journal of Epidemiology*.

